# Effect of Heat Treatment on Microstructural Evolution, Mechanical Properties, and Degradation Behavior of Zn-3Mg Alloy Fabricated by Laser Additive Manufacturing

**DOI:** 10.3390/mi17010007

**Published:** 2025-12-20

**Authors:** Changjun Han, Zhilang Chen, Hongtian Liu, Cheng Deng, Zhi Dong, Cheng Chen, Jinmiao Huang, Yongqiang Yang, Di Wang

**Affiliations:** 1 School of Mechanical and Automotive Engineering, South China University of Technology, Guangzhou 510641, China; cjhan@scut.edu.cn (C.H.); 202421001613@mail.scut.edu.cn (Z.C.); lht20021219@foxmail.com (H.L.); medongzhi@mail.scut.edu.cn (Z.D.); 202410180182@mail.scut.edu.cn (C.C.); 202120100525@mail.scut.edu.cn (J.H.); meyqyang@scut.edu.cn (Y.Y.); 2 College of Mechatronics Engineering, Guangdong Polytechnic Normal University, Guangzhou 510635, China

**Keywords:** zinc–magnesium alloy, laser powder bed fusion, annealing, triply periodic minimal surface, degradation

## Abstract

The Zn-3Mg alloy fabricated by laser powder bed fusion (LPBF) additive manufacturing is widely used in biomedical implants due to its excellent biocompatibility and favorable mechanical strength. However, its application is hindered by limited ductility and a relatively rapid degradation rate. This study investigated the influence of annealing heat treatment on the microstructure, mechanical properties, and degradation behavior of LPBF-fabricated Zn-3Mg porous implants. A systematic analysis of various annealing parameters revealed the evolution mechanisms of the microstructure, including grain coarsening and the precipitation and distribution of secondary phases Mg_2_Zn_11_ and MgZn_2_. The results indicated that appropriate annealing conditions (such as 250 °C for 1 h) significantly enhanced the compressive strain by 10%, while maintaining a high compressive strength of 24.72 MPa. In contrast, excessive annealing temperatures (e.g., 365 °C) promoted the formation of continuous brittle phases along grain boundaries, leading to deterioration in mechanical performance. The degradation behavior analysis illustrated a substantial increase in the corrosion rates from 0.6973 mm/year to 1.00165 mm/year after annealing at 250 °C for 0.5 h and 365 °C for 1 h, which can be attributed to the micro-galvanic effect induced by the presence of fine or coarse secondary phases that promoted localized corrosion. This study demonstrated synergistic regulation of mechanical properties and degradation behavior in the Zn-3Mg porous structures through optimized heat treatment, thereby providing essential theoretical and experimental supports for the clinical application of biodegradable zinc-based implants.

## 1. Introduction

Biodegradable metallic materials have demonstrated great potential in biomedical applications such as implantable medical devices, due to their unique biocompatibility and biodegradability [1,2]. Among these materials, zinc (Zn), magnesium (Mg), and iron alloys are the primary focus of research and development [3,4,5]. Compared to magnesium and iron alloys, Zn alloys exhibit a more moderate degradation rate, enabling a suitable in vivo service duration [6,7,8,9]. Notably, their degradation does not produce hydrogen gas, which is a significant advantage over Mg-based systems [10,11,12]. Furthermore, Zn is an essential trace element in the human body and plays a critical role in cell growth and tissue development. Zinc-magnesium (Zn-Mg) alloys are produced by incorporating Mg into a Zn matrix, thereby enhancing the mechanical strength of the matrix while simultaneously improving biocompatibility and osteogenic activity. Through the synergistic interaction between Zn and Mg, these alloys achieve a balanced combination of superior biocompatibility, controllable degradation kinetics, optimized mechanical properties, uniform corrosion behavior, and tunable functional performance [13,14,15]. Consequently, Zn-Mg alloys demonstrate significant promise for use in various applications, including bone regeneration, vascular stenting, and trauma fixation [16,17,18].

Laser powder bed fusion (LPBF) additive manufacturing can fabricate components through the layer-by-layer melting of metallic powders using a high-energy laser beam. This capability renders LPBF particularly suitable for the high-precision fabrication of porous Zn-Mg alloy implants [19,20,21,22,23,24]. The ultra-fast solidification process inherent in LPBF, characterized by cooling rates exceeding 10^6^ °C/s, promotes the formation of fine cellular and columnar sub-grains (<1 μm) in Zn-Mg alloys, which are significantly finer than those obtained through conventional casting processes (typically around 30 μm) [25,26]. Consequently, LPBF-fabricated Zn-Mg alloys exhibit markedly enhanced mechanical strength compared to their traditionally processed counterparts [27,28]. Voshage et al. [29] fabricated Zn-*x*Mg (*x* = 1, 2, 5 wt%) alloys with relatively high Mg content using LPBF. Their results indicated that as Mg content increased, the alloys became increasingly brittle, with fracture elongation decreasing and tensile strength declining from 381 MPa to 64 MPa. Similarly, Qin et al. [13] prepared Zn-Mg alloys with densities up to 99.5% by pre-alloying Zn-*x*Mg (*x* = 1, 2 and 5 wt%) powders. They observed that increasing Mg content led to higher hardness but progressively reduced plasticity. Their findings demonstrated that Mg addition contributed to grain refinement and promoted the formation of brittle intermetallic phases, thus impairing the ductility of the alloy. Yang et al. [30] studied the mechanical properties of Zn-*x*Mg (*x* = 1–4 wt%) prepared by LPBF and found that the Zn-3Mg alloy exhibited the highest tensile strength (reaching 220 MPa) with an elongation of 7.2%. During the LPBF fabrication of Zn-3Mg implants, intense temperature gradients and rapid solidification kinetics commonly induce significant residual stresses and non-equilibrium microstructures. These microstructural characteristics can compromise both degradation stability and long-term mechanical integrity. Although LPBF-processed Zn-3Mg alloy demonstrates enhanced biocompatibility and mechanical strength, its limited ductility, reflected in low elongation, remains a critical challenge.

Heat treatment is an effective method to improve the low plasticity of Zn-3Mg alloy fabricated by LPBF [31,32,33]. Han et al. [34] employed annealing treatment for the LPBF-fabricated Zn-1Mg alloy at 300 °C for 0.5 h, resulting in a decrease in tensile strength from 254.92 to 170.93 MPa, while elongation increased significantly from 0.55% to 8.43%. Dannbatta et al. [35] proposed an initial heat treatment process route for as-cast Zn-3Mg alloy, in which annealing at 370 °C for 10 h led to a more uniform microstructure and reduced casting defects. Notably, the tensile strength of the Zn-3Mg alloy decreased from 104 MPa to 88 MPa after annealing, whereas the elongation improved from 2.3% to 8.8% [36]. However, existing studies predominantly concentrate on the heat treatment of bulk Zn-Mg alloys, while limited attention has been given to porous Zn-Mg implant structures. Furthermore, these works did not elaborate on the underlying mechanisms for ductility improvement and failed to establish correlations between microstructural characteristics and performance. In conclusion, current understanding remains limited regarding the microstructure evolution of LPBF-fabricated Zn-3Mg alloy implants during heat treatment, as well as the synergistic regulation of their mechanical properties and degradation performance.

Therefore, this study aims to investigate the effect of annealing heat treatment on the microstructure, mechanical properties, and degradation performance of Zn-3Mg implants fabricated by LPBF, with particular emphasis on their deformation behaviors, to achieve optimal regulation of the strength-toughness synergy and degradation performance. Three typical triply periodic minimal surface (TPMS) porous structures (Primitive, Gyroid and Diamond) were used as porous implant architectures to identify the configuration offering superior printability and mechanical performance. The microstructure of LPBF-fabricated Zn-3Mg after annealing treatment was analyzed, and the effect of annealing on microstructure and its correlation with mechanical and degradation behaviors was discussed. This work will provide a valuable theoretical foundation and experimental guidance for the development of high-performance Zn-3Mg alloy implants.

## 2. Materials and Methods

### 2.1. Structural Design

**Figure 1:**
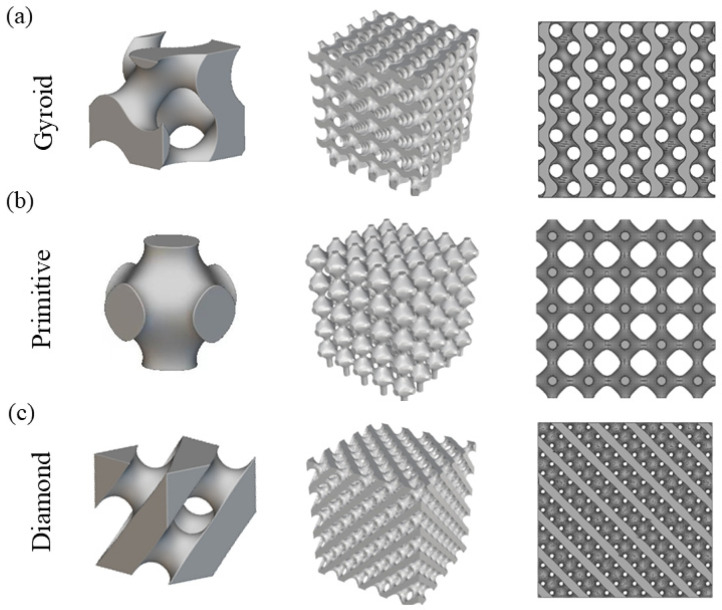
Model design of three types of TPMS unit cells along with their corresponding three-dimensional porous structures and top-view morphologies: (**a**) Gyroid, (**b**) Primitive, and (**c**) Diamond.

The TPMS structures enable dual mechanical and biological compatibility with human bone due to their unique biomimetic porous architectures, making them promising candidates for next-generation high-performance implants [37,38,39]. Among them, Gyroid, Primitive, and Diamond, which are three typical TPMS structures, exhibit significant advantages in the field of biomedical implants. They integrate highly interconnected pore structures, excellent mechanical properties, and favorable printability. Moreover, their high specific strength and large specific surface area enable their adaptation to complex geometric configurations. Additionally, these structures facilitate cell migration, nutrient transport, bone tissue ingrowth, and metabolic exchange [40,41,42]. Therefore, these three typical TPMS structures were selected to be designed and then fabricated using LPBF and systematically evaluated for surface morphology and mechanical properties under varying process parameters. Figure 1a shows the unit cells of the three TPMS structures, each measuring 2 mm × 2 mm × 2 mm. Figure 1b presents the structure models of different TPMS types, designed with dimensions of 10 mm × 10 mm × 10 mm and a porosity of 70%.

### 2.2. Preparation of Raw Materials

**Figure 2:**
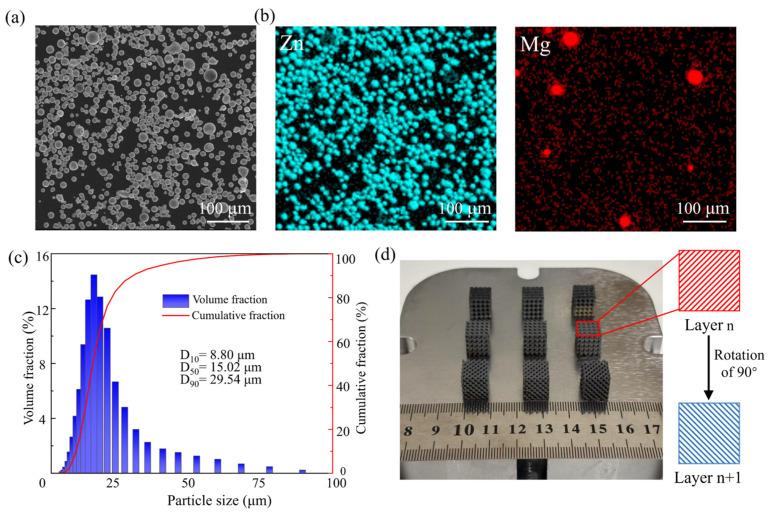
(**a**) SEM image showing the Zn-3Mg powder morphology; (**b**) EDS characterization of Zn and Mg elements; (**c**) particle size distribution of powder; (**d**) LPBF-fabricated Zn-3Mg structures.

The Zn-3Mg powder used in this experiment was mechanically mixed and provided by Shanghai Naiou Technology Co., Ltd., Shanghai, China. Figure 2a shows the morphology of the Zn-3Mg powder using a scanning electron microscope (SEM, FEI Nova nano 430, Eindhoven, The Netherlands), revealing a predominantly spherical shape, smooth surface, and minimal satellite particles, which indicate favorable powder morphology and excellent fluidity. Figure 2b presents the energy dispersive X-ray spectrometer (EDS) mapping of the Zn-3Mg powder, where the blue-green regions correspond to Zn and the red regions to Mg. The distribution of Mg particles appears uniform within the Zn matrix, suggesting homogeneous elemental dispersion. Particle size analysis was conducted using a LA960S laser particle size analyzer (HORIBA, Kyoto, Japan). The results indicated an average particle size of 15.02 μm, with D10 and D90 values of 8.80 μm and 29.54 μm, respectively (Figure 2c).

The LPBF experiment was performed using the Dimetal-100 machine (Laseradd, Guangzhou, China) equipped with an infrared ytterbium-doped fiber laser with a maximum laser power of 200 W, a maximum scanning speed of 7.8 m/s, and a maximum build envelope of 100 mm × 100 mm. Table 1 lists a set of LPBF parameters, including laser power, scanning speed, hatch space, and layer thickness. The volumetric energy density Ev was calculated according to Equation (1).

(1) $$E_{v}=\frac{P}{D_{s}\times H_{s}\times V}$$

where *P*, *V*, *D*_s_, and *H*_s_ represent laser power (W), scanning speed (mm/s), layer thickness (μm), and hatch space (μm), respectively. As shown in Figure 2d, the Zn-3Mg TPMS structure was prepared using a bidirectional orthogonal scanning strategy with an initial angle of 45° and a 90° rotation between consecutive layers. This scanning strategy effectively reduced thermal stress accumulation and improved sample quality. In addition, to minimize oxidation, high-purity argon gas was introduced as a shielding atmosphere during the printing process.

### 2.3. Annealing Heat Treatment

**Figure 3:**
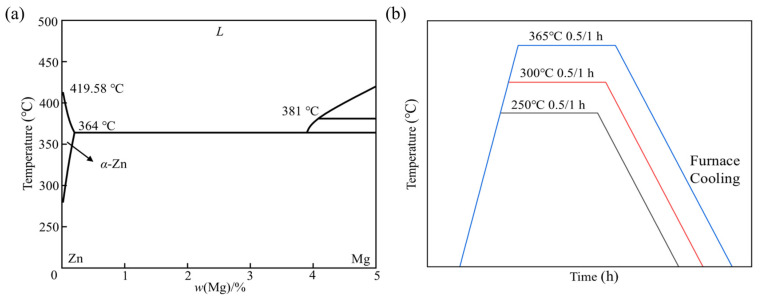
(**a**) Binary phase diagram of Zn-Mg alloy [34]; (**b**) heat treatment path.

Annealing heat treatment process was employed for the LPBF-fabricated Zn-Mg TPMS structures. Based on experimental results and equilibrium calculations of phase fractions, the LPBF-fabricated Zn-Mg alloys mainly consist of α-Zn, Mg_2_Zn_11_ and MgZn_2_ phases, which is consistent with the phase composition typically observed in conventional cast Zn-Mg alloys. Figure 3a shows the binary phase diagram of Zn-Mg alloys at low Mg concentrations. It can be seen that the solubility of Mg in the Zn matrix was highly limited (0.16 wt% at 364 °C and only 0.008 wt% at room temperature), which promoted the formation of Mg_2_Zn_11_ intermetallic phase even at low Mg contents. The diagram further revealed a eutectic point at approximately 3 wt% Mg, where the following eutectic reaction occurred:

(2) $${\mathrm{MgZn}}_{2}+L={\mathrm{Mg}}_{2}{\mathrm{Zn}}_{11}$$

The temperature for the formation of Mg_2_Zn_11_ ranged from 354.3 °C to 367.2 °C. However, this eutectic reaction was suppressed when the Mg content in the Zn-Mg alloy was below 3 wt%. To investigate this behavior, six short-term annealing treatment experiments were conducted in this study, as illustrated in Figure 3b. The annealing heat treatment was conducted using a Model GJC-SJ300 vacuum sintering furnace (Zhuzhou Guangjichang Technology Co., Ltd., Zhuzhou, China). For each annealing process, the heating rate was maintained at 10 °C/min, followed by furnace cooling. Based on the annealing temperature and holding time, the samples that have undergone different heat treatments are defined as 250 °C/300 °C/365 °C/0.5 h/1 h.

### 2.4. Charaterization

Prior to micro characterization, the LPBF-fabricated Zn-Mg structures were first immersed in industrial ethanol solution, and an ultrasonic cleaner was used to remove impurities (e.g., cutting fluid and dust) from the sample surfaces. Subsequently, the samples were ground with sandpapers of 400 #, 1000 #, 2000 #, and 3000 # grit sizes, respectively; when switching to a sandpaper with a higher grit size, the samples were rotated by 90°. Thereafter, the samples were subjected to rough polishing and fine polishing using Al_2_O_3_ and SiO_2_ polishing slurries, respectively. Finally, the polished surfaces were etched with a nitric acid-ethanol solution (4 wt% concentration) for approximately 10 s, followed by drying, thus completing the sample preparation.

Phase identification of the as-built and annealed Zn-3Mg structures was performed using a PANalytical X-ray polycrystalline diffractometer (XRD, Malvern Panalytical, Shanghai, China). The forming quality, microstructure, and corrosion products of the as-fabricated and heat-treated Zn-Mg samples were observed using SEM.

### 2.5. Mechanical Testing

A CMT5105 universal testing machine (SANS Corporation, Ningbo, China) was employed to evaluate the compressive properties of as-built and annealed Zn-3Mg TPMS structures, following the ASTM E9-09 standard [43]. Before compression testing, the porous structures were ultrasonically cleaned in an anhydrous ethanol solution for 20 min to remove surface contaminants such as residual powder and cutting fluid. The compression testing was conducted at 1 mm/min terminated at a displacement of 7 mm, corresponding to a nominal strain of 70%. Three replicate samples of each type of the Zn-3Mg TPMS structure were tested to ensure reliable and statistically significant results.

### 2.6. Immersion Test

The degradation performance test of the LPBF-built Zn-3Mg structure samples was conducted in accordance with the ASTM-G31-72 standard [44]. In vitro degradation tests were conducted in simulated body fluid (SBF) maintained at 37 °C and pH of 7.4 to mimic the physiological environment of the human body. Prior to testing, the Zn-3Mg sample surface was ground with 400# sandpaper to remove the oxide layer, followed by sequential polishing using 1000#, 2000#, and 3000# sandpaper to prepare a uniform surface for observing degradation products. The initial mass of each polished sample was measured using an electronic balance and recorded as *W*_0_. The dimensions of all surfaces were measured with a Vernier caliper, and the total surface area (mm^2^) was calculated accordingly. The SBF solution was prepared at a ratio of 10 mm of sample surface area per 1 mL of solution and sealed in test tubes for immersion testing over periods of 7, 14, 21, and 28 days. After each interval, corrosion products were observed using SEM coupled with EDS. The phase composition of the corrosion products was further determined by XRD. To remove surface corrosion products, samples were immersed in a cleaning solution containing CrO_3_ (200 g/L) and AgNO_3_ (10 g/L), then dried and reweighed to obtain the post-degradation mass, *W*_1_. The degradation rate of the Zn-3Mg sample was subsequently calculated according to Equation (3).

(3) $$D_{R}=8.76\times 10^{4}\times \frac{W_{0}-W_{1}}{\rho \times A\times t}$$

where *W*, *ρ*, *A*, and t denote the weight loss (g), the density of the zinc-magnesium alloy (g/cm^3^), the surface area (cm^2^), and the immersion time (h), respectively.

### 2.7. Electrochemical Test

The electrochemical behavior of Zn-3Mg structure samples in SBF was evaluated using a three-electrode system (Shanghai Chenhua Instrument Co., Ltd.,Shanghai, China), consisting of a platinum electrode, a reference electrode, and the alloy sample as the working electrode. Prior to testing, the surface oxide layer of the cubic Zn-3Mg samples was removed by grinding. The samples were then electrically connected using conductive adhesive applied to the bottom surface, followed by cold mounting in epoxy resin. After curing, the top surface was polished to expose a uniform cross-section for electrochemical analysis. The open-circuit potential (OCP) was monitored for 30 min until it stabilized dynamically. Finally, potentiodynamic polarization curves were recorded, and the corrosion potential (*E*_corr_) and corrosion current density (*I*_corr_) were determined via Tafel extrapolation.

## 3. Results and Discussion

### 3.1. Morphology and Mechanical Properties of As-Built Zn-3Mg Structures

The geometric differences in TPMS structures directly affect the interaction mechanism between the laser and materials. Variations in surface curvature, equivalent wall thickness gradients, and specific surface areas across different TPMS structures (e.g., Gyroid, Primitive, Diamond) lead to distinct processing behaviors. The Gyroid structure presents a smooth hyperbolic surface, while the Primitive structure features sharp edge transitions, resulting in localized energy concentration during laser scanning, particularly at regions with abrupt curvature changes, which may cause over-melting. Additionally, an increased specific surface area raises the risks of powder adhesion and incomplete melting [37,41]. Before fabricating Zn-3Mg porous structures via LPBF, single-track experiments and solid Zn-3Mg sample preparation were conducted. Under laser power ranging from 60 W to 90 W and scanning speeds of 600 mm/s to 900 mm/s, the single tracks demonstrated significantly improved continuity, with minimal discontinuities caused by unmelted powder, and the relative density of solid Zn-3Mg samples exceeded 90% [34]. Considering the markedly different responses of the three TPMS structures to laser energy input, this study established tailored process parameter sets for each structure and conducted comparative experiments to identify the optimal parameter combinations.

**Figure 4:**
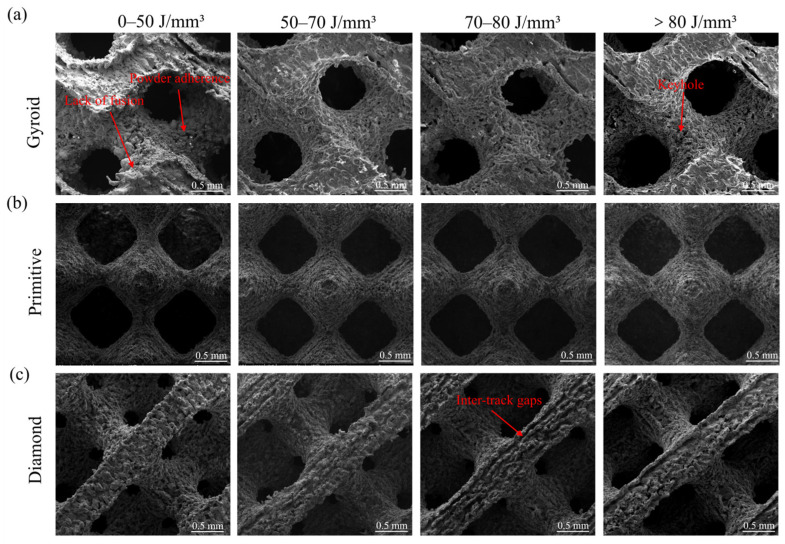
Surface morphologies of porous Zn-3Mg structures under different laser energy densities: (**a**) Gyroid, (**b**) Primitive and (**c**) Diamond.

Figure 4 shows the SEM images of the Zn-3Mg TPMS structures fabricated by LPBF. It is evident from the figure that variations in energy input parameters led to significant differences in surface morphology across the samples. When the laser energy density was lower than 50 J/mm^3^, multiple fractured and discontinuous melt tracks appeared at the junctions of the unit cells in all three TPMS structures, resulting in a rough and uneven surface. At a laser power of 60 W, increasing the scanning speed reduced the energy density, leading to insufficient laser energy absorption by the material. This caused the incomplete melting of the Zn-3Mg powder, increased adhesion of unmelted particles, and concurrent shrinkage and cracking of the melt tracks. When the laser energy density was increased to the optimal range of 50–70 J/mm^3^, the molten pool received a moderate amount of energy, enabling complete powder melting and the formation of continuous and uniform scanning tracks. The surface of the porous sample showed no obvious pores, indicating effective interlayer metallurgical bonding, improved surface quality, and enhanced relative density. However, when the laser energy density was further raised to 70–80 J/mm^3^, unmelted powder and gap defects reappeared on the surface, displaying random distribution and variable sizes. When the energy density exceeded 80 J/mm^3^, no significant adhered powder or gap defects were observed on the surface of the porous structures, suggesting complete melting of the powder. Nevertheless, excessive energy input promoted the accumulation of Zn vapor and entrapment of protective gas, which induced pore formation within the molten pool and ultimately deteriorated the fabrication quality [45,46].

At the same energy density, a significant gap between melted tracks was observed on the surface of the Diamond structure. This phenomenon arose from the relatively slender unit cells of the Diamond structure, which facilitated faster heat conduction and resulted in a higher cooling rate of the melt pool. Consequently, adjacent melt tracks failed to fully coalesce during solidification, thus leading to the formation of intertrack gaps. In addition, the scanning path employed for the Diamond structure was inherently complex. Under conditions of non-uniform energy density distribution, this complexity increased the likelihood of localized under-melting or over-melting, further promoting gap formation. As energy density increased, the extent of melt track separation initially worsened before gradually diminishing, while surface powder adhesion decreased. However, excessively high energy density induced abnormal protrusions around pore regions and generated spatters, compromising structural uniformity. Among the three TPMS structures examined, micro-porosity caused by excessive energy was observed. This was attributed to the relatively low boiling point of the Zn-3Mg alloy. High energy density led to excessive energy absorption, leading to over-melting. When the molten pool temperature exceeded a critical threshold, intense vaporization and evaporation of the Zn-3Mg alloy disturbed molten pool stability. By considering pore-related defects in the porous structure, surface quality, and the potential for under-melting or over-melting of the powder, the optimal laser energy density should be maintained within the range of 50–70 J/mm^3^. At a laser power of 70 W, complete melting of the Zn-3Mg powder was achieved across all three TPMS structures without excessive power input. Among them, when the scanning speed is 700–800 mm/s, all Zn-3Mg structures exhibited the best forming quality. The optimal combination was determined to be a laser power of 70 W and a scanning speed of 700 mm/s.

The mechanical properties of porous implants are strongly influenced by their structural design, porosity and manufacturing quality. However, even with identical porosity, TPMS structures exhibit significant differences in mechanical performance. To investigate the structure-dependent mechanical properties under optimized process parameters, compressive tests were carried out on the Zn-3Mg TPMS structures fabricated by LPBF. The compressive stress-strain curves of the three TPMS structures are presented in Figure 4a. It is evident that the Zn-3Mg structures exhibited relatively low ductility, with fracture occurring at strains below 4%. For all three TPMS structures, the compressive stress increased rapidly when the strain was below 3%, followed by a sharp decline, indicating fracture-induced deformation at this stage. The stress rise rate of the Zn-3Mg Gyroid structure was higher than that of the Diamond and Primitive structures, suggesting that the Gyroid structure possessed the highest elastic modulus among the three. Furthermore, the compressive stress of the Gyroid structure was significantly greater than that of Diamond and Primitive.

**Figure 5:**
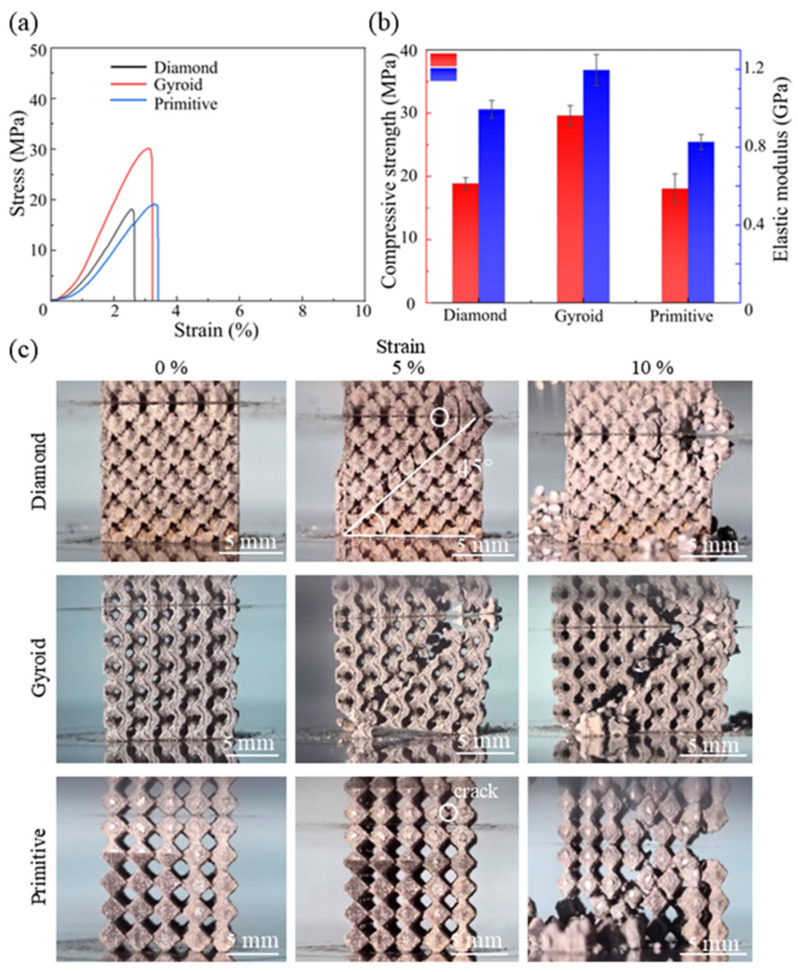
Compression properties and behaviors of LPBF-fabricated Zn-3Mg TPMS structures: (**a**) Stress-strain curves; (**b**) diagrams of compressive strength and elastic modulus; and (**c**) deformation behaviors of the three structures at different strains.

As shown in Figure 5b, the compressive strengths of Diamond, Gyroid and Primitive were 18.87 ± 0.93 MPa, 29.61 ± 1.60 MPa, and 18.07 ± 2.32 MPa, respectively, while their corresponding elastic modulus were 0.99 ± 0.05 GPa, 1.20 ± 0.08 GPa, and 0.83 ± 0.04 GPa, respectively. Figure 5c illustrates the compression deformation patterns of the three TPMS structures. It can be seen that during the initial deformation stage, failure initiated at the intercellular junctions. This occurred because these nodes underwent high compressive stress, leading to stress concentration, localized deformation, and subsequent cracking, which then propagated through the structure. Notably, at strains of 5% and 10%, all three TPMS structures exhibited fracture paths oriented along the 45° diagonal. This indicated that the fracture proceeded along the direction of maximum shear stress, presenting a shear-dominated deformation. The LPBF-fabricated Zn-3Mg Gyroid structure demonstrated the highest elastic modulus and compressive strength, making it a promising candidate for load-bearing scaffold applications.

### 3.2. Microstructure Evolution of Zn-3Mg Structures via Annealing

**Figure 6:**
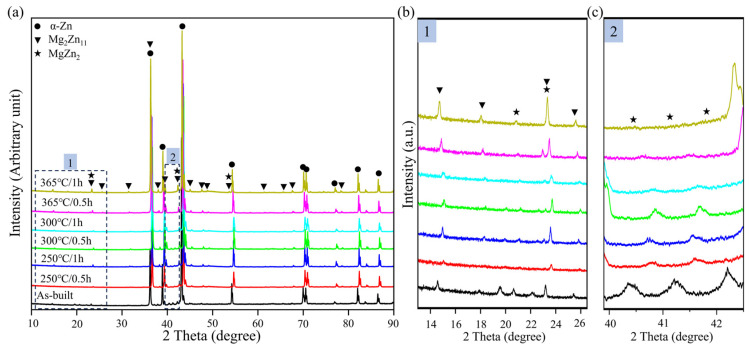
XRD patterns of the Zn-3Mg Gyroid structure prepared by LPBF before and after annealing: (**a**) Full-range profiles of α-Zn, MgZn_2_, and Mg_2_Zn_11_ phases; (**b**) Magnified profiles at 2θ = 10–30°; (**c**) Magnified profiles at 2θ = 40–43°.

The Zn-3Mg Gyroid structure fabricated using a laser power of 70 W and a scanning speed of 700 mm/s exhibits superior compressive performance, which is thus applied to annealing heat treatment. Figure 6 displays the XRD analysis of the LPBF-fabricated Zn-3Mg structure before and after annealing treatment. The diffraction peaks corresponding to the hexagonal close-packed crystal structure of α-Zn were observed in all samples, regardless of heat treatment conditions. No oxide-related peaks were detected in the XRD patterns, indicating minimal oxidation reaction occurred. Notably, at a diffraction angle of 14°, the Mg_2_Zn_11_ peak in sample 365 °C/1 h exhibited the highest intensity. Furthermore, under the identical temperature condition, an extended annealing time resulted in increased peak intensity. This phenomenon can be attributed to the fact that 365 °C was close to the eutectic temperature, where atomic diffusion was most efficient. A one-hour holding time allowed sufficient diffusion of Mg atoms, promoting their precipitation from the supersaturated α-Zn matrix and facilitating complete nucleation and grain growth. In contrast, heat treatments conducted at lower temperatures or shorter durations resulted in insufficient atomic diffusion, thereby retaining metastable phases within the microstructure of Zn-3Mg structure.

As shown in Figure 6b, after annealing, the MgZn_2_ peak near a diffraction angle of 20° disappeared due to the elimination of distortion-induced strengthening, accompanied by a significant reduction in peak intensity. Notably, the peak near 23° represented an overlapping contribution from both MgZn_2_ and Mg_2_Zn_11_ phases, and its increased intensity indicated that the precipitation of these two strengthening phases was simultaneously enhanced during heat treatment at 365 °C. At diffraction angles ranging from 40° to 43°, a consistent shift of the MgZn_2_ diffraction peaks toward higher angles is observed in all heat-treated samples relative to the as-built condition (Figure 6c). This shift can be attributed to the rapid solidification inherent in LPBF, which leads to supersaturation of Mg atoms within the α-Zn matrix. Such supersaturation induces lattice expansion and distortion, resulting in increased interplanar spacing. According to Bragg’s law, this increase causes the diffraction peaks of the as-fabricated microstructure to appear at lower angles. Subsequent heat treatment facilitates the precipitation and desaturation of excess Mg atoms from the matrix, thereby relieving lattice strain and restoring the crystal structure, which results in a shift of the diffraction peaks to higher angles.

**Figure 7:**
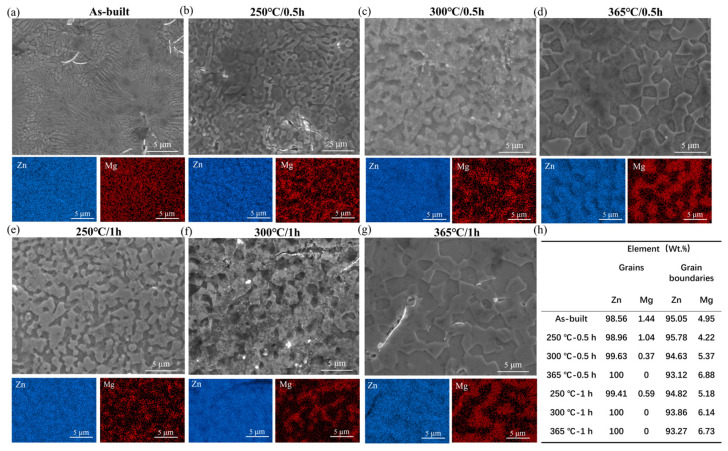
Microscopic morphologies and elemental distributions of Zn-3Mg structures with and without annealing: (**a**) as-built, (**b**) 250 °C/0.5 h, (**c**) 300 °C/0.5 h, (**d**) 365 °C/0.5 h, (**e**) 250 °C/1 h, (**f**) 300 °C/1 h, (**g**) 365 °C/1 h and (**h**) Zn and Mg element contents at different positions.

The morphology and corresponding elemental distribution of the Zn-3Mg structure fabricated by LPBF are shown in Figure 7. As illustrated in Figure 7a–d, the grain size of the Zn-3Mg alloy increased with the increase in annealing temperature. Compared with the as-built structure (with an average grain size of 0.5 μm), the structures annealed for 0.5 h at 250 °C, 300 °C, and 365 °C exhibited a continuous increase in average grain size, which ultimately reached approximately 4 μm. Annealing treatment enhanced atomic diffusion, facilitating the transformation of the microstructure from a metastable state to a thermodynamically stable configuration, primarily through grain coarsening. In the as-built Zn-3Mg alloy, the fine-grained structure resulted in a relatively high density of grain boundaries. Following annealing at 250 °C, grain growth occurred, accompanied by the precipitation and segregation of supersaturated Mg atoms to the grain boundaries. The second phase appeared in a discontinuous and particulate form. EDX mapping confirmed the existence of α-Zn and secondary phases, with the latter predominantly localized at grain boundaries. Quantitative analysis of EDS spectra revealed that with increasing annealing temperature, the Mg concentration at grain boundaries rose from 4.95 wt% in the as-built structure to 6.88 wt%, while the intragranular Mg content decreased from 1.44 wt% to nearly 0 wt% (Figure 7h). This indicated progressive Mg depletion within grains and its enrichment at boundaries, leading to the formation of continuous or semi-continuous network-like secondary phases.

As depicted in Figure 7e–g, in the Zn-3Mg alloy structure annealed for 1 h, the Mg element was predominantly enriched at the grain boundaries. With increasing annealing time, the Mg concentration at the grain boundaries gradually increased, whereas the Mg content within the grains correspondingly decreased. The measured grain sizes of the structures annealed for 1 h at 250 °C, 300 °C, and 365 °C were approximately 2–3 μm, 2.5–4 μm, and 3–5 μm, respectively, indicating progressive grain coarsening compared to the Zn-3Mg structure annealed for 0.5 h. At a constant temperature, prolonged holding duration enhanced the mobility of grain boundaries, enabling them to migrate toward thermodynamically more stable configurations, while residual Mg atoms were allowed to diffuse toward the grain boundaries. Extended annealing promoted microstructural evolution, which significantly impacted the strength and ductility of the structure. Following high-temperature annealing at 365 °C, pronounced grain coarsening was observed, along with nearly complete depletion of Mg from the grain interiors. This indicated extensive diffusion of Mg atoms to the grain boundaries, where they precipitated to form a coarse and continuous network of the MgZn_2_ and Mg_2_Zn_11_ secondary phase. The formation of the Mg_2_Zn_11_ phase was further corroborated by the increased peak intensity in the XRD patterns. Notably, the presence of such secondary phase along the grain boundaries introduced a structural weakness. Due to its inherent brittleness, this network acted as a preferential site for crack initiation and facilitated rapid crack propagation under applied stress. Therefore, it is considered the primary factor responsible for the severe deterioration in both strength and ductility, ultimately leading to material embrittlement after annealing at 365 °C.

### 3.3. Mechanical Properties

**Figure 8:**
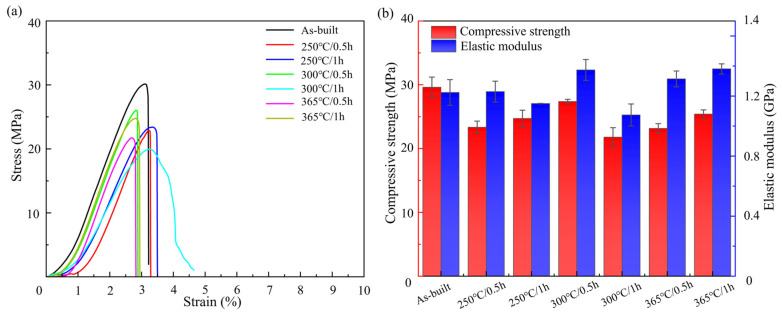
Compression properties of LPBF- fabricated Zn-3Mg structures before and after annealing treatment: (**a**) Stress-strain curves; and (**b**) compressive strength and elastic modulus.

Figure 8 shows the mechanical properties of the LPBF-fabricated Zn-3Mg porous structures with and without annealing. The as-built Zn-3Mg scaffold exhibited a compressive strength of 29.61 ± 1.60 MPa, an elastic modulus of 1.15 ± 0.01 GPa, and a compressive strain of 3.13 ± 0.02%. Annealing treatment led to a moderate reduction in compressive strength, while the elastic modulus of 1.2 GPa remained largely unchanged. Following annealing, the compressive strength decreased by up to 26.43%. The Zn-3Mg porous structures annealed at 250 °C for 0.5 h, 250 °C for 1 h, and 300 °C for 1 h showed a more gradual increase in compressive stress during the initial stage of compression, indicating enhanced plastic deformation ability. This suggested improved structural integrity retention and greater compressive strain pr ior to fracture. At 250 °C, both compressive strength and strain increased with prolonged holding time. Specifically, after annealing for 1 h, the Zn-3Mg scaffold achieved a compressive strength of 24.72 ± 1.40 MPa and a compressive strain of 3.46 ± 0.01%, representing 83.49% of the as-built compressive strength while showing an approximately 10% improvement in elongation. The LPBF-fabricated as-built samples contained a large number of fine grains and a supersaturated α-Zn matrix due to rapid solidification. After annealing, the grains coarsened to a moderate degree. However, the concurrently precipitated, finely-dispersed MgZn_2_/Mg_2_Zn_11_ secondary phases exert a dispersion-strengthening effect, which compensated for a portion of the strength loss caused by grain coarsening, resulting in only a slight decrease in strength. In addition, annealing at 250 °C effectively alleviated the residual stress generated during the LPBF fabrication process. Meanwhile, the finely-dispersed secondary phases did not form a continuous brittle network and thus did not hinder dislocation slip. Instead, they enhanced the plasticity of the structure by coordinating grain deformation.

At an annealing temperature of 300 °C, the mechanical properties of the Zn-3Mg scaffold exhibited significant variation with holding time. After 1 h of annealing, the compressive strength decreased to 21.78 ± 1.40 MPa, representing a 26.41% reduction compared to the as-built condition, while the compressive strain increased by only 5.5%. At 365 °C, annealing for 0.5 h and 1 h resulted in compressive strengths of 23.16 ± 0.75 MPa and 25.41 ± 0.67 MPa, and elastic modulus of 1.31 ± 0.05 GPa and 1.38 ± 0.03 GPa, respectively. When the annealing temperature exceeded the optimal range, heat treatment adversely affected the mechanical performance of the Zn-3Mg scaffold. This mainly stems from excessive grain coarsening and the continuous network-like precipitation of brittle secondary phases. Therefore, an optimized heat treatment process can effectively improve the compressive strain while preserving the compressive performance of the LPBF-fabricated Zn-3Mg scaffold.

### 3.4. Degradation Properties

**Figure 9:**
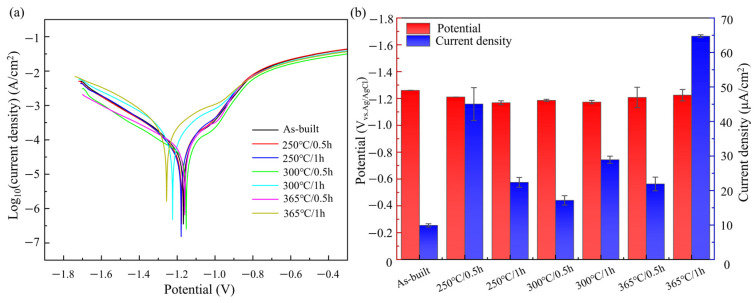
Electrochemical characteristics of LPBF-fabricated Zn-3Mg structures before and after annealing treatment: (**a**) Polarization curves; and (**b**) corrosion potential and corrosion current density.

The degradation behavior of a material is primarily governed by electrochemical corrosion, a key mechanism underlying its degradation process. Electrochemical techniques can be used to assess the instantaneous corrosion rate, which is particularly indicative of the material’s initial degradation tendency. To investigate the effect of annealing treatment on the degradation performance of LPBF-fabricated Zn-3Mg structures, electrochemical tests were conducted on structures in both the as-built and heat-treated conditions. Figure 9a shows the potentiodynamic polarization curves of LPBF-fabricated Zn-3Mg alloy before and after annealing. Distinct passivation behaviors were observed between the as-built structure and the various heat-treated conditions. This phenomenon can be attributed to the transition of the structure surface from rapid dissolution to the formation of a protective passive film during anodic polarization in the Zn-3Mg alloy. Compared to the as-built state, the porous Zn-3Mg structure after heat treatment exhibits a delayed inflection point, which can be attributed to the finer grain structure possessing a larger specific surface area than its coarse-grained counterpart. During anodic polarization, the refined microstructure provides a greater number of active sites, potentially resulting in a higher initial anodic dissolution rate.

The corrosion potential (E_corr_) of the Zn-3Mg porous structure annealed at 250 °C for 0.5 h decreased from −1.26 ± 0.001 V to −1.168 ± 0.015 V compared to the as-built structure. A lower corrosion potential indicated a relatively higher thermodynamic stability and a reduced tendency for spontaneous dissolution. However, after annealing, the corrosion current density increased under all treatment conditions, indicating an accelerated corrosion rate. Specifically, the corrosion current densities of the 250 °C/0.5 h and 365 °C/1 h samples reached 45.05 ± 4.76 μA/cm^2^ and 64.72 ± 0.42 μA/cm^2,^ respectively, significantly exceeding the as-built structure value of 9.89 ± 0.46 μA/cm^2^. For the structure annealed at 250 °C for 0.5 h, fine MgZn_2_ and Mg_2_Zn_11_ phases precipitated from metastable clusters and were widely distributed on the structural surface, acting as cathodic sites that promoted cathodic reactions [47]. Meanwhile, large areas of the α-Zn matrix remained exposed, facilitating the formation of efficient micro-galvanic couples that accelerated corrosion. In contrast, annealing at 365 °C for 1 h led to the formation of coarse second phases on the surface, which exhibited a larger electrochemical potential difference relative to the matrix, thereby inducing intense localized galvanic corrosion. The corrosion rate (R) can be calculated according to the following equation [34]:

(4) $$R=3.27\times 10^{-3}\frac{I_{corr}}{\rho }m_{e}$$

where *ρ* and *m_e_* are the densities (g/cm^3^) of the Zn-3Mg alloy and its equivalent metal mass, respectively. For Zn-3Mg, *ρ* is determined to be 6.5319 g/cm^3^ and *m_e_* is 32.4847. Among the various annealing conditions, the degradation rates of 250 °C/1 h, 300 °C/0.5 h, and 365 °C/0.5 h samples fell within the ideal degradation rate range (0.2–0.5 mm/year) for biodegradable alloys. Notably, the degradation rate of sample 250 °C/1 h increased from 0.1531 ± 0.0001 mm/year in the as-built structure to 0.34635 ± 0.02186 mm/year after annealing. These results indicated that annealing accelerated the degradation of the Zn-3Mg structure.

**Figure 10:**
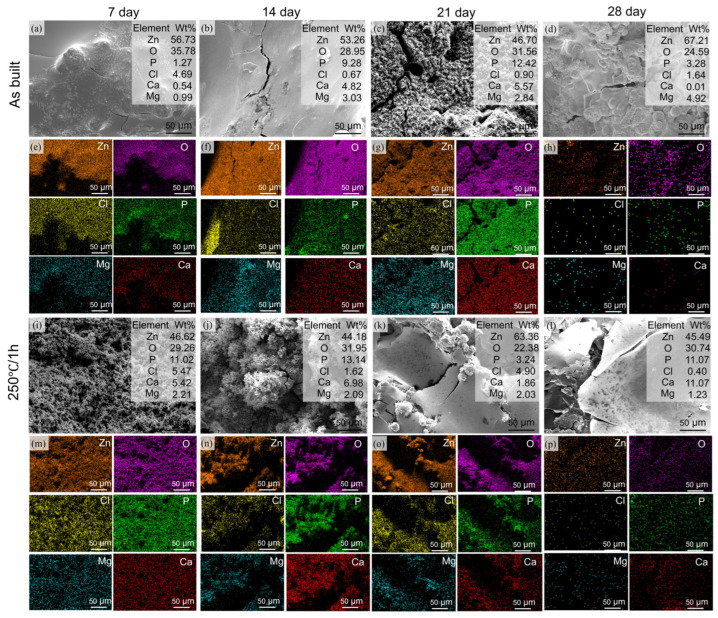
Degradation products of LPBF-fabricated Zn-3Mg structures after immersion for 7, 14, 21, and 28 days: (**a**–**h**) as-built state; (**i**–**p**) 250 °C/1 h.

To fully understand the long-term degradation behavior of the Zn-3Mg structures before and after annealing treatment under service conditions, including the evolution of corrosion products and their protective effect, immersion tests over extended periods are indispensable. Therefore, in vitro degradation experiments were conducted on both the as-built and annealed (250 °C/1 h). After 28 days of immersion testing, the weight losses of the as-built and 250 °C/1 h samples were measured as 0.1213 ± 0.0002 g and 0.2079 ± 0.0013 g, respectively. Based on Equation (3), their corresponding corrosion rates were calculated to be 0.146 mm/year and 0.251 mm/year. Figure 10 illustrates the microstructural evolution and formation mechanism of degradation products on the surfaces of the as-built and 250 °C/1 h samples. At the initial stage of degradation, a smooth corrosion layer formed on the surface of the as-built structure. As degradation proceeded, this layer gradually thickened and developed fine microcracks. With continued exposure, localized spallation occurred, followed by the deposition of flocculent and loosely packed corrosion products. Eventually, a relatively uniform grayish-white protective layer was reformed on the surface. In contrast, the surface of the 250 °C/1 h sample generated a large amount of loose and porous flocculent deposits during the initial degradation stage, which rapidly aggregated into snowflake-like morphologies. These aggregates subsequently deposited to form smooth shell-like and cellular patterns, accompanied by extensive cracking. Ultimately, thick yet loosely compacted sheet-like films were formed.

Since the inorganic salts and other components in the SBF solution can chemically react with Zn and Mg ions, EDS was used to identify the degradation products. The results indicated the presence of Zn, Mg, Ca, Cl, and P in the degradation layer, with their chemical forms and relative contents differing from those observed in sample 250 °C/1 h. At the initial stage of degradation, Zn^2+^ ions released from the anode reaction reacted with Cl^−^ ions in the SBF solution, leading to the uniform precipitation of hydrozincite on the surface [34]. This formation process can be described by the following equations:

(5) $$\mathrm{Zn}+2H_{2}O\to {\mathrm{Zn}}^{2+}+H_{2}+2{\mathrm{OH}}^{-}$$

(6) $$Zn^{2+}+2{\mathrm{OH}}^{-}\to \mathrm{ZnO}+H_{2}O$$

(7) $$5Z\mathrm{nO}+2{\mathrm{Cl}}^{-}+6H_{2}O\to {\mathrm{Zn}}_{5}(\mathrm{OH}{)}_{8}{\mathrm{Cl}}_{2}H_{2}O+2{\mathrm{OH}}^{-}$$

(8) $$5\mathrm{Zn}(\mathrm{OH}{)}_{2}+2{\mathrm{HCO}}_{3}^{-}+2H^{+}\to {\mathrm{Zn}}_{5}({\mathrm{CO}}_{3}{)}_{2}(\mathrm{OH}{)}_{6}+4H_{2}O$$

As the reaction proceeded, the accumulation of OH^−^ ions increased the local pH value. The SBF solution contained buffering species such as bicarbonate and hydrogen phosphate ions, which helped stabilize the system. Under these conditions, some smithsonite transformed locally into more stable phases, including hydroxyapatite and basic zinc carbonate, which adhered to the surface of the corrosion layer [30,48]. Concurrently, hydrogen gas generated during the cathodic reaction accumulates beneath the dense layer, creating internal pressure that eventually causes cracking and spalling of the deposit. The fine grain size and homogeneous microstructure of the as-built Zn-3Mg phase facilitated a relatively uniform re-passivation process during degradation [49,50].

Compared to the as-built state, the Zn-3Mg structure annealed at 250 °C for 1 h underwent a continuous and accelerated corrosion process. This severe corrosion preferentially initiated at grain boundaries. After annealing for 1 h at 250 °C, a large number of fine and dispersed MgZn_2_ and Mg_2_Zn_11_ phases (cathodic phases) are precipitated, which form a vast quantity of uniformly distributed micro-galvanic couples with the α-Zn matrix (anodic phase). As efficient cathodes with lower potentials, these MgZn_2_ and Mg_2_Zn_11_ phases promoted intense micro-galvanic coupling, leading to the rapid release of substantial amounts of Zn^2+^ and Mg^2+^ ions [51]. The subsequent hydrolysis of these metal ions, combined with hydrogen evolution at the cathode, induced a sharp increase in pH at the corrosion interface. Consequently, PO_4_^3−^ and Ca^2+^ ions in SBF rapidly reached supersaturation and precipitated extensively on the structural surface as amorphous calcium phosphate and zinc phosphate, as described by

(9) $${\mathrm{HPO}}_{4}^{2-}\to {\mathrm{PO}}_{4}^{3-}+H^{+}$$

(10) $${9\mathrm{Ca}}^{2+}+6{\mathrm{PO}}_{4}^{3-}\to {\mathrm{Ca}}_{9}{{(\mathrm{PO}}_{4})}_{6}$$

(11) $${3\mathrm{Zn}}^{2+}+{2\mathrm{PO}}_{4}^{2-}+{2\mathrm{OH}}^{-}\to {\mathrm{Zn}}_{3}{{(\mathrm{PO}}_{4})}_{2}+2H_{2}O$$

This precipitation occurred abruptly, with a disordered molecular arrangement that prevented the formation of a compact structure, ultimately yielding a loose, porous morphology characterized by flocculent and snowflake-like aggregates. The formation of amorphous calcium phosphate and zinc phosphate occurred simultaneously, resulting in a mixed deposit composed of zinc-calcium phosphate. This amorphous zinc calcium phosphate formation is attributed to the release of Mg^2+^ from the annealing-induced secondary phase, which interferes with the ordered arrangement of Ca^2+^ and PO_4_^3−^ ions during the inhibition of phosphate crystallization, thereby promoting the formation of an amorphous structure [52]. The amorphous structure in the Zn-3Mg porous structure tends to incorporate a significant amount of water molecules within its lattice, leading to volumetric expansion and reducing the overall density of the deposit. Furthermore, the coexistence of Ca^2+^, Zn^2+^, and Mg^2+^ ions exerted a synergistic inhibitory effect on crystallization, suppressing long-range structural ordering and thereby promoting the development of a highly disordered and porous architecture. Due to its lack of structural integrity, this deposit was unable to form an effective protective barrier between the Zn-3Mg surface and the corrosive environment, which accounted for the accelerated corrosion rate observed in the 250 °C/1 h sample.

## 4. Conclusions

This study systematically investigated the role of annealing heat treatment on the microstructure, mechanical properties, and degradation behavior of Zn-3Mg porous implants fabricated by laser powder bed fusion (LPBF). The main conclusions are as follows:

Annealing heat treatment effectively promoted the equilibrium transformation of metastable microstructure in LPBF-fabricated Zn-3Mg alloy. Mg atoms precipitated from the supersaturated α-Zn matrix and formed MgZn_2_ and Mg_2_Zn_11_ secondary phases at grain boundaries. High-temperature and long-time heat treatment (such as 365 °C for 1 h) caused the coarsening of the phases and the formation of a continuous network structure, while medium- and low-temperature, short-duration treatment (such as 250 °C for 1 h) resulted in fine and uniformly dispersed secondary phases.

Heat treatment has a pronounced effect on the mechanical behavior of the LPBF-fabricated Zn-3Mg structures. The 250 °C/1 h sample preserved a high compressive strength of 24.72 MPa and simultaneously improved compressive strain by approximately 10%, demonstrating an effective balance between strength and plasticity. In contrast, excessively high annealing temperatures promoted the precipitation of coarse and continuous brittle phases, which acted as preferential sites for crack initiation and propagation, thereby compromising structural integrity and leading to a significant deterioration in overall mechanical performance.

Compared with the as-built state, the degradation rate of the 250 °C/1 h sample increased to 0.251 mm/year from 0.146 mm/year, yet remained within the ideal degradation range of 0.2–0.5 mm/year. The accelerated degradation originated from the fine second phases acting as efficient cathodes, promoting uniform microgalvanic corrosion. The degradation products evolved from dense chlorozincite (Simonkolleite) in the as-built state to a loose, amorphous mixed deposit layer composed of zinc-calcium-phosphate, leading to the continuous and rapid corrosion.

## Figures and Tables

**Table 1:** Laser powder bed fusion (LPBF) process parameters for printing Zn–3Mg structures.

Parameters	Value
Laser power (W)	60, 70, 80, 90
Scanning speed (mm/s)	600, 700, 800, 900
Hatch space (μm)	55
Layer thickness (μm)	30

## Data Availability

The data presented in this paper are available on request from the corresponding author. The data are not publicly available due to privacy restrictions.

## Abbreviations

The following abbreviations are used in this manuscript:

LPBF	Laser Powder Bed Fusion
TPMS	Triply Periodic Minimal Surface
SEM	Scanning Electron Microscope
EDS	Energy Dispersive X-ray Spectrometer
XRD	X-Ray polycrystalline Diffractometer
SBF	Simulated Body Fluid
OCP	Open-Circuit Potential
